# Winter is coming: hibernation reverses the outcome of sperm competition in a fly

**DOI:** 10.1111/jeb.12792

**Published:** 2015-12-28

**Authors:** P. Giraldo‐Perez, P. Herrera, A. Campbell, M. L. Taylor, A. Skeats, R. Aggio, N. Wedell, T. A. R. Price

**Affiliations:** ^1^ Institute of Integrative BiologyUniversity of LiverpoolLiverpoolUK; ^2^ Division of Microbial EcologyUniversity of ViennaViennaAustria; ^3^ Centre for Ecology and ConservationBiosciencesCollege of Life and Environmental SciencesUniversity of ExeterCornwallUK; ^4^ Institute of Translational MedicineUniversity of LiverpoolLiverpoolUK

**Keywords:** *Drosophila pseudoobscura*, long‐term sperm storage, meiotic drive, overwintering, paternity share, polyandry, selfish genetic element, sperm competition, sperm storage

## Abstract

Sperm commonly compete within females to fertilize ova, but research has focused on short‐term sperm storage: sperm that are maintained in a female for only a few days or weeks before use. In nature, females of many species store sperm for months or years, often during periods of environmental stress, such as cold winters. Here we examine the outcome of sperm competition in the fruit fly *Drosophila pseudoobscura*, simulating the conditions in which females survive winter. We mated females to two males and then stored the female for up to 120 days at 4°C. We found that the outcome of sperm competition was consistent when sperm from two males was stored for 0, 1 or 30 days, with the last male to mate fathering most of the offspring. However, when females were stored in the cold for 120 days, the last male to mate fathered less than 5% of the offspring. Moreover, when sperm were stored long term the first male fathered almost all offspring even when he carried a meiotic driving sex chromosome that drastically reduces sperm competitive success under short‐term storage conditions. This suggests that long‐term sperm storage can radically alter the outcome of sperm competition.

## Introduction

Females of most species are polyandrous, mating with more than one male (Taylor *et al*., 2014). In many of these species, sperm from multiple males compete within the female to fertilize her eggs. Sperm competition can have major impacts on the evolution of reproductive traits in males and females, investment in mating and reproduction, conflict within families and the sexes and on the evolution of cooperation (Birkhead & Møller, 1998; Hughes *et al*., 2008; Pizzari & Wedell, 2013). Researchers have used controlled laboratory studies to examine the mechanisms of sperm competition, such as visualizing sperm movements inside the female as competition occurs (e.g. Manier *et al*., 2010), and using applied molecular techniques to assign paternity in natural populations (e.g. Rodríguez‐Muñoz *et al*., 2010). However, the majority of sperm competition studies have only examined sperm competition in the short term, with sperm stored in the female for only a few days or weeks before the ova are fertilized (Pearse *et al*., 2001).

In nature, females of many species store sperm for long periods of time, sometimes well beyond the timescale of a single breeding cycle (Birkhead & Møller, 1993). Mammals, fish, reptiles, amphibians, birds, gastropods and insects have all been observed to store sperm for months or years (Birkhead & Møller, 1993; Holt & Lloyd, 2010), although in many cases parthenogenesis has not been completely ruled out (Booth & Schuett, 2011). Molecular techniques have confirmed sperm storage durations of decades in social insect queens (Boomsma *et al*., 2005), five years in snakes (Booth & Schuett, 2011), three years in turtles (Pearse *et al*., 2001) and three years in fish (Bernal *et al*., 2014). Many species become inactive during harsh periods, such as hibernation over winter (e.g. Almeida‐Santos & Salomào, 1997; Collett & Jarman, 2001) or aestivation over hot or dry periods (e.g. mosquitoes: Lehmann *et al*., 2010), and in many species, females carry sperm through these periods. At present, we have no idea how the duration of long‐term sperm storage relates to sperm competitive success. If the sperm and ejaculate traits required for successful long‐term sperm storage are different to those required for success in sperm competition, long‐term sperm storage could fundamentally alter the outcome of sperm competition. In this case, sperm and ejaculate traits may have evolved in response to the needs for long‐term sperm storage and sperm competitive success, rather than the short‐term sperm competitive success typically measured in the laboratory. However, the impact of long‐term storage on sperm competitive outcomes has only been examined in long‐lived species that are difficult to study in the laboratory, such as large‐colony social insects and marine reptiles (Birkhead & Møller, 1993; Holt & Lloyd, 2010). Moreover, most of the studies have been observational (Uller & Olsson, 2008), analysing the paternity of the broods of wild or occasionally captive females. Whereas this approach has demonstrated that long‐term sperm storage occurs, it is impossible to know how many males mated with a female but fathered no offspring, or how many matings and ejaculates each female received from each male. This lack of data has limited our ability to unravel the direct impacts of long‐term sperm storage on sperm competitive success.

Here we investigate the impact of over‐winter sperm storage on sperm competitive success in *Drosophila pseudoobscura*, a naturally polyandrous (Price *et al*., 2011) fly found in forests in western North and Central America from Guatemala to Canada (Dobzhansky & Epling, 1944). *D. pseudoobscura* are inactive at temperatures below 11°C (Dobzhansky & Epling, 1944) and cannot survive more than a few days at temperatures below 0°C (Crumpacker & Marinkovic, 1967). However, they overwinter as adults (Dobzhansky & Epling, 1944), taking refuge in locations that do not freeze, as do all other members of the *Obscura* group (Lumme & Lakovaara, 1983). A laboratory study found that male *D. pseudoobscura* are far more susceptible to low temperatures than females, with fewer males surviving extended periods of cold exposure than females (Collett & Jarman, 2001). Moreover, many males that survived the cold were rendered sterile. In locations with long winters, it is therefore possible that all males die over winter, and the first new generation each spring is entirely fathered using sperm that was carried over winter by females. Here we use females from Show Low, Arizona, a location which experiences daily maximum temperatures below 10°C from mid‐November to mid‐March, and daily maximum temperatures below 6°C from December through February (National Climatic Data Center, USA).

To allow us to determine the paternity of different males, we used the selfish meiotic driving X chromosome *sex ratio* or *SR* as a genetic marker. We chose *SR* rather than a visible mutant marker such as *Sepia* or *Vermillion*, because *SR* is found at far higher frequencies in nature (Jaenike, 2001) and is less impaired at sperm competition (Wu, 1983; Price *et al*., 2008), making *SR* more biologically relevant. The alternative allele to *SR* is the nondriving X chromosome, which is generally referred to as *standard* (*ST*). In males, *SR* causes the developmental failure of all sperm carrying Y chromosomes (Policansky & Ellison, 1970; Beckenbach, 1981), and more than 95% of the sperm produced by *SR* males carry the *SR* X chromosome (Cobbs *et al*., 1991; Beckenbach, 1996). The remaining sperm carry neither the X nor Y and result in the production of pseudo‐males, that is infertile, XO individuals. It is not clear whether meiotic drive increases the rate of production of XO pseudomales, or if it simply reveals the underlying rate of XO pseudomales in normal *Drosophila* (e.g. Cobbs *et al*., 1991), but pseudomales are seen in many *Drosophila* species (e.g. Stern & Hadorn, 1938), often in studies of meiotic drive (Cazemajor *et al*., 2000). Further, in some species, XO males are fertile, and may also represent resistance against the extinction effect of drive (Voelker & Kojima, 1972). The production of XO pseudomales by XY drive bearing males is typically rare (< 2% of offspring).

Here we investigate whether sperm competitive success is affected by long‐term sperm storage by females over winter.

## Materials and methods

### Fly stocks

We collected wild female *D. pseudoobscura* in July 2012 at Show Low, Arizona (34°07037″ N; 110°07037″ W). We pooled offspring from the wild females to produce a mass population (*N* > 200 each generation). We maintained this for one year before experiments were conducted in 2013. We created the mass population using standard (‘ST’) flies (i.e. flies that did not carry the *SR* meiotic driver). We also collected a strain of *SR* from the site, confirmed by examining the proportion of sons produced (< 1%) and a PCR assay (described below). We maintained this *SR* strain as a mass population by crossing *ST* males into the *SR* population at each generation. This will have resulted in the genotypes from the mass population being introgressed into the *SR* mass population. Hence, after 12 generations, the *SR* mass population is expected to have been genetically identical to the *ST* mass population, except that all X chromosomes are *SR* chromosomes. This strain of *SR* produces less than 1% pseudo‐male offspring.

We kept flies in standard *Drosophila* vials (25 ×75 mm) on a medium of rolled oats, brown sugar, dried yeast, agar, nipagin and water (Shorrocks, 1972), and maintained them at 23°C under a 14:10 h photoperiod, with lights on at 10:00 GMT. We transferred flies by aspiration and did not anaesthetize them as this is known to disrupt copulation behaviour (Barron, 2000) and male fertility (Champion de Crespigny & Wedell, 2006).

### Sperm competition trials

We collected female virgins from the *ST* populations and males from both *ST* and *SR* populations. We separated flies by sex within 18 h of eclosion to ensure virginity. We kept males in groups of 10, separated by genotype. Females were kept in groups of 10. At 3 days old, we placed individual females into a separate vial and allowed them to acclimatize overnight. At 4 days old, we aspirated a male into each vial and watched for 3 h to observe copulation, with the experiment starting at 10:00 GMT. We discarded any pairs that failed to copulate. When pairs copulated, we removed and discarded the male. Four days later, we presented each mated female with a second 4‐day‐old virgin male and observed any second copulations. Any eggs laid by females between the two matings were discarded. We discarded females that failed to copulate a second time (61%). The first mate of each female was either an *ST* or *SR* male. Females mated first to an *ST* male were then remated to an *SR* male, and vice versa. Hence, we gave every female one mate that was *SR* and one that was *ST*. Observations were conducted ‘blind’ using different people to set up the matings and observe them and by labelling vials with an uninformative number to prevent any potential observer bias.

### Cold treatment

To simulate Arizona winters and cold fronts, at 9 days old, we randomly assigned each twice‐mated female to a cold treatment lasting 0, 1, 30 or 120 days. Usually, in Show Low, Arizona, winter lasts 3‐4 months (120 days); 30 days simulates cold fronts, usually seen in April and the short winters endured by low altitude populations in some Arizona desert borders; 1 day simulates a cold night, which occurs often and also provides a test of whether any impact of cold is due simply to cold shock, or to duration in the cold; and 0 days is the control (temperature information from the US National Oceanic and Atmospheric Association). Females given the 0 days cold treatment were simply moved onto a new vial. Following Collett & Jarman (2001) females in the other treatments were moved to a refrigerator and kept in the same vial at 4°C for either 1, 30 or 120 days in complete darkness to simulate natural conditions of being buried under leaf litter, bark, etc. We maintained humidity above 0% to prevent flies dying from desiccation. After their respective cold treatment, we moved each female to a new vial kept at 23°C and allowed her to oviposit. We removed females that had died during the cold treatment from the experiment. We moved all females to a new vial every 3 days, for a total of 9 days of oviposition at 23°C. We pooled progeny from the three vials of each female and sexed the offspring, to give the proportion of sons produced. Where females produced more than 100 offspring, we sexed only 100 randomly selected offspring (18% of females produced more than 100 offspring, mean offspring number was 65). Whereas male offspring could only have been fathered by the *ST* male, female offspring could have been fathered by either male. To determine paternity of offspring, we randomly selected 23 daughters and genotyped them for *SR*, with appropriate controls, using a previously described PCR assay (Price *et al*., 2011). We standardized to this number because it was the smallest number of daughters produced by a female. We extracted DNA using the ‘fly squish’ method (Gloor *et al*., 1993). Single flies were squashed with a cocktail stick in 50 *μ*L buffer (10 mm Tris‐Cl @pH 8.2, 1 mm EDTA, 25 mm NaCl and 200 *μ*g mL^−1^ freshly diluted Proteinase K). These were then incubated at ~35°C for 25 min followed by a further incubation at 95°C for 1.5 min. Samples were spun and kept in the fridge prior to PCR. We then used PCR amplification of the *SR* diagnostic gene using 10 pmol *μ*L^−1^ DPSSR primers to genotype females (method described in Price *et al*., 2011). PCR products were determined using gel electrophoresis on a 2.5% agarose gel with 3 *μ*L Midori green per 100 mL of TAE buffer. Each well was loaded with 5 *μ*L of PCR product diluted with 3 *μ*L loading dye, and we used Bioline Hyperladder V for amplicon size determination. Gels were run at 120V for ~30 min and photographed. Offspring counts and genotyping were conducted ‘blind’ using the original vial numbers used in the mating trials.

### Data analysis

We analysed the proportion of females that produced offspring after the cold treatment using a generalized linear model (GLM) with binomial error structure and a probit link function. The order of mating and the duration in the cold were included in the maximal model as fixed factors. We simplified the maximal model by the stepwise removal of nonsignificant factors and levels. We then examined the differences between the four cold durations using two‐tailed *Z*‐tests.

We estimated the proportion of offspring fathered by the *SR* male by multiplying the proportion of daughters produced by the proportion of daughters that carried *SR*. We then used two types of analyses. Firstly, we used a very simple nonparametric analysis to investigate the impact of mating order and cold duration on the proportion of offspring fathered by the *SR* male. We then used a more complex GLM to analyse the same data. If both simple and complex analyses produced similar results, this would give us strong confidence in our results. For our first, simple analysis, we used Kruskal–Wallis and Wilcox test to determine whether the genotype of the first male to mate increased or decreased the proportion of *SR* bearing offspring, for each cold duration. Kruskal–Wallis and Wilcox test were applied, as the data were not normally distributed, and could not be transformed to normality, due to being skewed around 0% for some conditions, and 100% for others.

In a second analysis, we used GLM to more accurately determine the impact of mating order and the number of days spent in the cold on the proportion of *SR* in the offspring, and to test for interactions between cold duration and mating order. For this, we used an R package called glmulti. In summary, the glmulti package performs an exhaustive screening of the candidate factors (e.g. days in the cold and genotype of the first male to mate) and reports the best model as the one associated with the smallest Akaike information criterion (AIC). In our study, the factors tested were the proportion of *SR* in the offspring as the *response* or dependent variable, and the genotype of the first male and the days spent in the cold as *terms* or independent variables (i.e. ProportionOfSR ~ GenotypeFirstMale * DaysInCold). Then, we used anova to compare the proposed best model vs. a different model where the genotype of the first male was not considered (i.e. ProportionOfSR ~ DaysInCold). A *P*‐value lower than 0.05 indicates that the removed factor (i.e. the genotype of the first male) is an important factor for the final proportion of *SR*. This analysis was also performed combining days 0 and 1, then combining days 0, 1 and 30. Finally, we reanalysed the data using glmulti, this time including total number of offspring produced by each female as a factor, to examine whether variation in female fecundity was skewing the analysis. All analyses were carried out using R version 3.1.2 (Ihaka & Gentleman, 1996).

## Results

The proportion of females that successfully produced offspring was significantly affected by duration in the cold (proportion that produced offspring: 0 days: 116/123; 1 day: 109/117; 30 days: 48/63; 120 days: 29/61; GLM *χ* ^2^ test: *χ* ^2^_3,365 _= 61.748, *P *<* *0.001; see Supplementary Table S1). Mating order (GLM *χ* ^2^ test: *χ* ^2^_3,364 _= 0.014, *P *=* *0.906), and the interaction between mating order and duration in the cold (GLM *χ* ^2^ test: *χ* ^2^_3,363 _= 1.183, *P *=* *0.277) had no significant impact on whether females produced offspring or not. Females kept in the cold for 0 or 1 day did not differ in their likelihood of producing offspring (*Z*‐test: *Z* = 0.367, *P *=* *0.711). However, significantly fewer females produced offspring after 30 days of exposure to cold than 0 or 1 days (*Z*‐test: *Z* = 4.153, *P *<* *0.001), and females kept cold for 120 days were least likely to successfully produce offspring (*Z*‐test comparing 30 and 120 days of cold: *Z* = 3.288, *P *<* *0.001). Further, the total number of offspring is a factor that affects the accuracy of our estimation of brood sex ratio. However, the number of offspring was relatively high, ranging from 41 to over 200. As we only sexed a maximum of 100 offspring, the difference in accuracy in sex ratio estimation is unlikely to have been large, at worst 41 vs. 100. More importantly, the number of offspring produced would have had no effect on the proportion of daughters that carried SR, as 23 daughters were consistently genotyped in every family.

**Figure 1:**
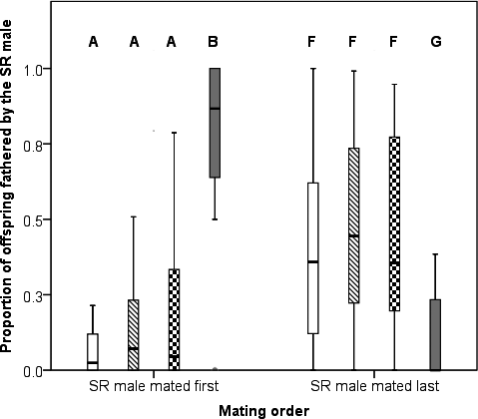
The proportion of offspring fathered by the *SR* male when a female mated to both an *SR* and an *ST* male. Following mating, females were stored at 4°C for 0 (white boxes), 1 (boxes with diagonal lines), 30 (checked boxes) or 120 days (grey boxes). Horizontal bar, box and whiskers indicate the median, interquartile range and range, respectively. Significant differences in offspring paternity between cold duration treatments within a mating order are indicated by the letters above the bars. Note that, this figure does not show the significance of differences across the two mating orders, so A differs from B, but is not directly compared here to F and G.

Ignoring all other factors, a lower median proportion of *SR* offspring were produced when the *SR* male mated first (*SR* first: Median = 0.052, *N* = 120, *SR* second: Median = 0.369, *N* = 120, *W *=* *10 428, *P *<* *0.001). Hence, in subsequent analyses, we split the data by mating order. When an *SR* male mated first, they fathered significantly more offspring after females spent 120 days cold than 0, 1 or 30 days (Fig. 1; Kruskal–Wallis test: *χ* ^2 ^= 11.660, d.f. = 3, *P* = 0.009;). However, when they mated second they fathered significantly fewer offspring after females spent 120 days cold than 0, 1 or 30 days (Fig. 1; Kruskal–Wallis test: *χ* ^2 ^= 8.946, d.f. = 3, *P* = 0.030;). These results were confirmed by the GLM analysis. The glmulti package reported that the best model would involve the genotype of the first male to mate, the days spent in the cold and the interaction between these two factors (i.e. ProportionOfSR ~ GenotypeFirstMale + DaysInCold + DaysInCold:GenotypeFirstMale). We used anova to compare this suggested model to a new model where the genotype of the first male to mate is not considered (i.e. ProportionOfSR ~ DaysInCold). The results indicate that the genotype of the first male to mate is an important factor affecting the proportion of *SR* in the offspring (d.f. = 2; Deviance = 5.53; *F* = 33.32; *P* < 0.001). We performed the same comparison, this time omitting from the suggested model the interaction between the genotype of the first male to mate and days in the cold, which returned similar results (d.f. = 1; Deviance=2.95; *F* = 31.53; *P* < 0.001). When we combined days 0 and 1; and days 0, 1 and 30, the glmulti package suggested the same best model (i.e. ProportionOfSR ~ GenotypeFirstMale + DaysInCold + DaysInCold:GenotypeFirstMale) and the genotype of the first male to mate was again reported as an important factor for the proportion of SR found in the offspring (Days 0 and 1: d.f. = 2; Deviance = 5.54; *F* = 33.43; *P* < 0.001, Days 0, 1 and 30: d.f. = 2; Deviance = 5.5; *F* = 33.05; *P* < 0.001).

To check that our analysis was robust, and not an artefact of our method of estimating the proportion of offspring fathered by the *SR* male (i.e. using the *SR* allele as a paternity marker), we repeated the analysis using only the proportion of sons produced. Finally, we repeated the analysis using only the proportion of genotyped daughters that were fathered by the *SR* male. Both these analyses were concordant with the original analysis, showing the same significant effects (results not shown). Further, in a parallel glmulti analysis, we included the number of offspring as a potential factor. As expected, the number of offspring had no significant effect on the proportion of *SR* offspring (d.f. = 1; Deviance = 0.01; *F* = 0.10; *P* = 0.753). Data are archived at Dryad (datadryad.org).

## Discussion

This experiment examined the outcome of sperm competition after females spent time in the cold, to simulate over‐winter sperm storage. At 0, 1 and 30 days, exposure to cold the proportion of offspring fathered by each male followed the pattern of last male sperm precedence previously observed in *D. pseudoobscura* (Wu, 1983; Price *et al*., 2008), and many other *Drosophila* and insects (Simmons, 2001). However, when females experienced 120 days of cold exposure, the first male to mate fathered almost all the offspring, irrespective of the male's genotype. Even *SR* males, that are typically disadvantaged in sperm competition, (Price *et al*., 2008), were able to father more than 95% of offspring if they were the first mate of a polyandrous female subsequently exposed to 120 days of cold. This suggests that when females experience long overwintering periods, this can radically affect the outcome of sperm competition.

There are two possible criticisms of this interpretation. The first is that sample sizes were relatively low for females that experienced 120 days of cold. Whereas the sample sizes were smaller for the 120‐day treatment, the change from the last male fathering most offspring in the 0‐30 day treatments, to the first male fathering almost all offspring in the 120 day treatment is unlikely to have occurred due to lower sample size. Small sample size is typically expected to increase variance, making differences nonsignificant, rather than drive two strongly significant results. Hence, the change in sperm competitive outcome at 120 days of cold exposure is unlikely to be due to chance combined with low sample sizes. The second criticism is that the experiment may not be a good model of what flies experience in the wild, as females were rapidly placed in cold conditions with no gradual change in temperature or light regime changes that might indicate the onset of winter. However, the flies were derived from populations in the central USA where the climate is continental, and so can experience a sudden drop in temperature and onset of winter with little warning (www.ncdc.noaa.gov).

If we assume that the experiment is a reasonable model of over‐winter sperm storage in these flies, then what mechanisms might drive the change in sperm competitive success following long‐term sperm storage? One possibility is that the females may not have had sufficient time to store sperm from the second male before they were placed in the cold, as some studies suggest that sperm storage in *Drosophila* can take up to two days (Schnakenberg *et al*., 2012). However, for this mechanism to drive the change in sperm competitive outcome seen at 120‐day cold exposures, a mechanism is required by which the sperm from the second mating was stored successfully for the 1‐ and 30‐day cold treatment flies, but not for the 120‐day flies, which seems unlikely.

An alternative possibility is that sperm from the first and second males may have been stored in different sperm storage organs, as has been suggested in dung flies (Otronen *et al*., 1997). *Drosophila* females typically have two types of sperm storage organ, the seminal receptacle and spermathecae (Pitnick *et al*., 1999), and sperm is stored in both after mating (Schnakenberg *et al*., 2012). The seminal receptacle is a long thin blind tubule extending off of the uterus, and its length is tightly linked to the length of the sperm produced by males across species in *Drosophila* (Pitnick *et al*., 1999). In *D. melanogaster*, the proportion of sperm in the seminal vesicle from a particular male strongly predicts the proportion of offspring that male will father (Manier *et al*., 2013). In contrast, the spermathecae are a pair of sperm storage organs that are highly chitinized round capsules, each connected to the uterus by a duct (Pitnick *et al*., 1999; Heifetz & Rivlin, 2010). The purpose of the spermathecae is not well understood. They are not essential for sperm storage, having been gained or lost in several *Drosophila* lineages (Pitnick *et al*., 1999), and evidence suggests they may have a secretory role as well as a sperm storage function (Schnakenberg *et al*., 2011). Exactly why there are two types of sperm storage organs in many *Drosophila* females is not clear (Schnakenberg *et al*., 2012). One possibility is that the spermathecae are long‐term sperm storage organs (Pitnick *et al*., 1999), and their tough exterior is to help protect stored sperm (Heifetz & Rivlin, 2010), whereas the seminal receptacle is a short‐term storage organ. Sperm are stored in both the spermathecae and seminal vesicle after mating in *D. pseudoobscura* (Snook *et al*., 1994), but this has only been examined in singly mated females. It is possible that ejaculates from additional matings are largely stored in the seminal receptacle. If sperm survive long‐term cold exposure in the spermathecae, but not the seminal receptacle, this may explain the change in the outcome of sperm competition following cold storage. Further, it is possible that the seminal fluids from both males may also act differently inside overwintering females. As some seminal fluid proteins act specifically to enhance success when a male mates first (sperm competition defence), whereas others increase success when a male mates with a nonvirgin female (sperm competition offence) (Avila *et al*., 2011), it is possible that defence proteins survive better in overwinter conditions, providing an advantage to the first male to mate. However, these possibilities will require further testing.

If long‐term sperm storage is generally associated with major changes in the outcome of sperm competition, then this has major implications. Firstly, effective population size is typically lowest during periods of environmental stress, such as winter (e.g. Begon, 1977) or hot or dry summers (e.g. Lehmann *et al*., 2010). Hence, the outcome of sperm competition during these long‐term sperm storage events might be particularly important for species inhabiting challenging environments. The extreme variation seen in sperm and sperm storage organ morphology in *Drosophila* (Pitnick *et al*., 1999) and other insects (Simmons, 2001) might be partially explained by differences in the pattern of long‐term sperm storage (e.g. aestivation vs. hibernation), and in contrasts between the relative importance of long and short‐term sperm storage. For example, in a hypothetical univoltine species in which only females overwinter, a long winter might strongly select for sperm that survive the cold well. In a multivoltine species on the other hand, in which only females overwinter, some generations will experience the warm season short‐term sperm competition that is typically studied, whereas only in the winter generation would long‐term cold sperm competition be important. Sperm and ejaculates are highly variable within and between species, and it is likely that this variation is maintained by trade‐offs (Lüpold *et al*., 2012). It is possible there may be trade‐offs in sperm traits for long‐term and short‐term sperm competition, with different populations potentially showing a different suite of sperm and ejaculate traits (Snook, 2005). For example, if sperm survival is key to long‐term sperm competitive success, this might select for a smaller number of higher quality sperm, contrasting with the prediction that sperm competition typically selects for increased sperm number (Snook, 2005; Simmons & Fitzpatrick, 2012). The transfer of accessory gland proteins that manipulate female reproductive physiology (Wolfner, 1997) might also be disadvantageous during long‐term sperm storage if they reduce the chance that the female will survive the winter, or affect her subsequent fecundity in the spring. Alternatively, males might detect the onset of colder weather and alter their spermatogenesis to produce sperm or ejaculates better adapted to the cold (Wigby *et al*., 2009; Price *et al*., 2012). However, the stresses experienced by sperm during long‐term storage do not have to be driven by cold temperatures. High temperatures are also particularly likely to damage stored sperm, and female‐mediated spermicide during sperm storage may also adversely affect sperm survival (Holman & Snook, 2008). Long‐term sperm competition may happen less frequently than short‐term competition, or it may be selected on in fewer generations. Indeed, the balance between the importance of long and short‐term sperm competition might be related to the number of generations the species has a year. For example, bivoltine species may often be more highly adapted to overwinter sperm competition, whereas in species with multiple summer generations, the frequent selection for warm temperature sperm competition performance might hinder adaptation to winter sperm storage conditions. This could produce an interesting example of intergenerational intralocus conflict, in which males cannot adapt fully to overwinter sperm competition because the same genes are also selected in response summer sperm competition. Alternatively, low winter population sizes might increase the influence of genetic drift, slowing adaptation. How species might alter their summer and winter sperm storage and sperm competition abilities in the face of climate change might be particularly interesting.

The small number of observational studies that have examined sperm competition in females that stored sperm long term have not found the extreme paternity bias towards the first male to mate that we find in cold exposed *D. pseudoobscura* (painted turtles, *Chrysemys picta*: Pearse *et al*., 2001; shiner perch, *Cymatogaster aggregata*; Liu & Avise, 2011). To our knowledge, the only other study to directly compare long‐ and short‐term sperm storage paternity patterns is in the Ocoee salamander (*Desmognathus ocoee*), where paternity outcomes were similar between salamanders that stored sperm for a few days before use and those that stored sperm for months (Adams *et al*., 2005). This suggests that patterns of sperm use of long‐term stored sperm, and any differences between long‐term and short‐term sperm competition, may be taxon specific. In 2003, Mack *et al*. published a study that assessed the impact of female age on sperm competition in three strains of *D. melanogaster* maintained at normal experimental temperatures. Although the oldest experimental females were 30 days old (as opposed to our 120‐day‐old females), an increase in offspring fathered by the first mate (P1) and reduction by the second mate (P2) was seen, as a consequence of increasing female age, with the greatest difference seen between the female ages of 3.5 and 17 days. The decrease in P2/P1 proportion we report after increased time in the cold follows Mack *et al*.'s trend, and so we cannot be certain that the change in paternity is simply due to cold rather than female age. In terms of biological relevance, this distinction may be moot as *D. pseudoobscura* females are unlikely to live more than three weeks in nature if they are not overwintering (Dobzhansky & Wright, 1943), so very old females will only be those that have overwintered.

The extremely high paternity gained by the first male when females were exposed to cold for four months is puzzling in light of the distribution and dynamics of *SR* in natural populations of *D. pseudoobscura*. *SR* is absent from Canada, and more common in the southern USA, and there is some evidence that *SR* is less common immediately after the end of winter (Dobzhansky & Epling, 1944; Price *et al*., 2014). Hence, we expected that long‐term exposure of a female to cold might reduce the success of *SR* males in sperm competition relative to *ST* males. However, the results contradict this prediction. At normal temperature, *SR* males fathered a mean of only 25% of offspring (14% when mated first and 36% when mated second, for a mean of 25%). However, when females overwintered, *SR* males’ mean fatherhood increased to 43% (74% when mated first, 12% when mated second). In other words, long‐term cold exposure of females appears to increase the success of *SR* males when mating to a virgin female, as they will father most of her offspring. In effect, our results show that females that overwinter utilize sperm in a similar manner to singly mated females. As *SR* X chromosomes are more successful than *ST* X chromosomes when females only mate once because there is no sperm competition to counteract the transmission advantage of *SR* drive (Price *et al*., 2010), *SR* should be more successful when females overwinter. Hence, over‐winter sperm storage is unlikely to explain the absence of *SR* from northern populations.

In conclusion, we show that when females experienced a long‐term cold period, this affects the outcome of sperm competition, with the first male to mate fathering almost all offspring, in contrast to the normal pattern of last male sperm precedence. This finding has implications for mating patterns, the evolution of sperm storage organs, and the success of selfish genes that manipulate spermatogenesis.

## Supporting information


**Table S1** Number of females surviving each temperature treatment, and the number that successfully produced offspring.

## References

[jeb12792-bib-0001] Adams, E. , Jones, A. & Arnold, S. 2005 Multiple paternity in a natural population of a salamander with long‐term sperm storage. Mol. Ecol. 14: 1803–1810. 15836651 10.1111/j.1365-294X.2005.02539.x

[jeb12792-bib-0002] Almeida‐Santos, S. & Salomào, M. 1997 Long‐term sperm storage in the female neotropical rattlesnake *Crotalus durissus terrificus* (Viperidae: Crotalinae). Japan J. Herpetol. 17: 46–52.

[jeb12792-bib-0003] Avila, F.W. , Sirot, L.K. , LaFlamme, B.A. , Rubinstein, C.D. & Wolfner, M.F. 2011 Insect seminal fluid proteins: identification and function. Ann. Rev. Entomol. 56: 21–40. 20868282 10.1146/annurev-ento-120709-144823 PMC3925971

[jeb12792-bib-0004] Barron, A.B. 2000 Anaesthetising *Drosophila* for behavioural studies. J. Insect Physiol. 46: 439–442. 12770207 10.1016/s0022-1910(99)00129-8

[jeb12792-bib-0005] Beckenbach, A.T. 1981 Multiple mating and the “sex‐ratio” trait in *Drosophila pseudoobscura* . Evolution 35: 275–281. 10.1111/j.1558-5646.1981.tb04886.x 28563371

[jeb12792-bib-0006] Beckenbach, A.T. 1996 Selection and the “sex‐ratio” polymorphism in natural populations of *Drosophila pseudoobscura* . Evolution 50: 787–794. 10.1111/j.1558-5646.1996.tb03888.x 28568936

[jeb12792-bib-0007] Begon, M. 1977 The effective size of a natural *Drosophila subobscura* population. Heredity 38: 13–18. 268311 10.1038/hdy.1977.2

[jeb12792-bib-0008] Bernal, M.A. , Sinai, N.L. , Rocha, C. , Gaither, M.R. , Dunker, F. & Rocha, L.A . 2014 Long‐term sperm storage in the brownbanded bamboo shark *Chiloscyllium punctatum* . J. Fish Biol. 86: 1171–1176. 25545440 10.1111/jfb.12606

[jeb12792-bib-0009] Birkhead, T.R. & Møller, A. 1993 Sexual selection and the temporal separation of reproductive events: sperm storage data from reptiles, birds and mammals. Biol. J. Linn. Soc. 50: 295–311.

[jeb12792-bib-0010] Birkhead, T.R. & Møller, A.P ., eds. 1998 Sperm Competition and Sexual Selection. Academic Press, San Diego.

[jeb12792-bib-0011] Boomsma, J. , Baer, B. & Heinze, J. 2005 The evolution of male traits in social insects. Ann. Rev. Entom. 50: 395–420. 10.1146/annurev.ento.50.071803.130416 15822204

[jeb12792-bib-0012] Booth, W. & Schuett, G. 2011 Molecular genetic evidence for alternative reproductive strategies in North American pitvipers (Serpentes: Viperidae): long‐term sperm storage and facultative parthenogenesis. Biol. J. Linn. Soc. 104: 934–942.

[jeb12792-bib-0013] Cazemajor, M. , Joly, D. & Montchamp‐Moreau, C. 2000 Sex‐ratio meiotic drive in *Drosophila simulans* is related to equational nondisjunction of the Y chromosome. Genetics 154: 229–236. 10628983 10.1093/genetics/154.1.229 PMC1460905

[jeb12792-bib-0014] Champion de Crespigny, F.E. & Wedell, N. 2006 *Wolbachia* infection reduces sperm competitive ability in an insect. Proc. R. Soc. B 273: 1455–1458. 10.1098/rspb.2006.3478 PMC1560322 16777737

[jeb12792-bib-0015] Cobbs, G. , Jewell, L. & Gordon, L. 1991 Male‐sex‐ratio trait in *Drosophila pseudoobscura*: frequency of autosomal aneuploid sperm. Genetics 127: 381–390. 2004709 10.1093/genetics/127.2.381 PMC1204365

[jeb12792-bib-0016] Collett, J. & Jarman, M. 2001 Adult female *Drosophila pseudoobscura* survive and carry fertile sperm through long periods in the cold: populations are unlikely to suffer substantial bottlenecks in overwintering. Evolution 55: 840–845. 11392402 10.1554/0014-3820(2001)055[0840:afdpsa]2.0.co;2

[jeb12792-bib-0100] Crumpacker, D.W. & Marinkovic, D. 1967 Preliminary evidence of cold temperature resistance in Drosophila pseudoobscara. Am. Nat. 101: 505–514.

[jeb12792-bib-0017] Dobzhansky, T. & Epling, C. 1944 Contributions to the Genetics, Taxonomy, and Ecology of Drosophila Pseudoobscura and Its Relatives. Carnegie Institution of Washington, Washington, DC.

[jeb12792-bib-0018] Dobzhansky, T. & Wright, S . 1943 Genetics of natural populations. X. Dispersal rates in *Drosophila pseudoobscura* . Genetics 28: 304e340. 17247091 10.1093/genetics/28.4.304 PMC1209213

[jeb12792-bib-0019] Gloor, G. , Preston, C. , Johnson‐Schlitz, D. , Nassif, N. , Phillips, R. , Benz, W. *et al* 1993 Type I repressors of P element mobility. Genetics 135: 81–95. 8224830 10.1093/genetics/135.1.81 PMC1205629

[jeb12792-bib-0020] Heifetz, Y. & Rivlin, P. 2010 Beyond the mouse model: using *Drosophila* as a model for sperm interaction with the female reproductive tract. Theriogenology 73: 723–739. 20015541 10.1016/j.theriogenology.2009.11.001

[jeb12792-bib-0021] Holman, L. & Snook, R.R. 2008 A sterile sperm caste protects brother fertile sperm from female‐mediated death in *Drosophila pseudoobscura* . Curr. Biol. 18: 292–296. 18291649 10.1016/j.cub.2008.01.048

[jeb12792-bib-0022] Holt, W. & Lloyd, R. 2010 Sperm storage in the vertebrate female reproductive tract: How does it work so well? Theriogenology 73: 713–722. 19632711 10.1016/j.theriogenology.2009.07.002

[jeb12792-bib-0023] Hughes, W. , Oldroyd, B. , Beekman, M. & Ratnieks, F. 2008 Ancestral monogamy shows kin selection is key to the evolution of eusociality. Science 320: 1213–1216. 18511689 10.1126/science.1156108

[jeb12792-bib-0024] Ihaka, R. & Gentleman, R. 1996 R: A language for data analysis and graphics. J. Comp. & Graph. Stat. 5: 299–314.

[jeb12792-bib-0025] Jaenike, J. 2001 Sex chromosome meiotic drive. Ann. Rev. Ecol. & Syst. 32: 25–49.

[jeb12792-bib-0026] Lehmann, T. , Dao, A. , Yaro, A. , Adamou, A. , Kassogue, Y. , Diallo, M. *et al* 2010 Aestivation of the African malaria mosquito, *Anopheles gambiae* in the Sahel. Am. J. Trop. Med. Hyg. 83: 601–606. 20810827 10.4269/ajtmh.2010.09-0779 PMC2929058

[jeb12792-bib-0027] Liu, J.‐X. & Avise, J. 2011 High degree of multiple paternity in the viviparous Shiner Perch, *Cymatogaster aggregata*, a fish with long‐term female sperm storage. Mar. Biol. 158: 893–901. 24391263 10.1007/s00227-010-1616-0 PMC3873152

[jeb12792-bib-0200] Lumme, J. & Lakovaara, S. 1983 Seasonality and diapause in Drosophilids. In: The Genetics and Biology of Drosophila, vol. 3d (Ashburner M., Carson H.L., Thompson J.N. Jr, eds), pp. 171–220. Academic Press, New York.

[jeb12792-bib-0028] Lüpold, S. , Manier, M.K. , Berben, K.S. , Smith, K.J. , Daley, B.D. , Buckley, S.H. *et al* 2012 How multivariate ejaculate traits determine competitive fertilization success in *Drosophila melanogaster* . Curr. Biol. 22: 1667–1672. 22840512 10.1016/j.cub.2012.06.059

[jeb12792-bib-0029] Mack, P.D. , Priest, N.K. & Promislow, D.E.L. 2003 Female age and sperm competition: last‐male precedence declines as female age increases. Proc. R. Soc. B 270: 159–165. 10.1098/rspb.2002.2214 PMC1691224 12590754

[jeb12792-bib-0030] Manier, M. , Belote, J. , Berben, K. , Novikov, D. , Stuart, W. & Pitnick, S. 2010 Resolving mechanisms of competitive fertilization success in *Drosophila melanogaster* . Science 328: 354–357. 20299550 10.1126/science.1187096

[jeb12792-bib-0031] Manier, M. , Lüpold, S. , Pitnick, S. & Starmer, W. 2013 An analytical framework for estimating fertilization bias and the fertilization set from multiple sperm‐storage organs. Am. Nat. 182: 552–561. 24021407 10.1086/671782

[jeb12792-bib-0032] Otronen, M. , Reguera, P. & Ward, P. 1997 Sperm storage in the yellow dung fly *Scathophaga stercoraria*: identifying the sperm of competing males in separate female spermathecae. Ethology 103: 844–854.

[jeb12792-bib-0033] Pearse, D. , Janzen, F. & Avise, J. 2001 Genetic markers substantiate long‐term storage and utilization of sperm by female painted turtles. Heredity 86: 378–384. 11488975 10.1046/j.1365-2540.2001.00841.x

[jeb12792-bib-0034] Pitnick, S. , Markow, T. & Spicer, G. 1999 Evolution of multiple kinds of female sperm‐storage organs in *Drosophila* . Evolution 53: 1804–1822. 10.1111/j.1558-5646.1999.tb04564.x 28565462

[jeb12792-bib-0035] Pizzari, T. & Wedell, N. 2013 The polyandry revolution. Phil. Trans. R. Soc. Lond. B. 368: 20120041. 23339233 10.1098/rstb.2012.0041 PMC3576576

[jeb12792-bib-0036] Policansky, D. & Ellison, J. 1970 “Sex ratio” in *Drosophila pseudoobscura*: spermiogenic failure. Science 169: 888–889. 5432586 10.1126/science.169.3948.888

[jeb12792-bib-0038] Price, T.A.R. , Bretman, A.J. , Avent, T.D. , Snook, R.R. , Hurst, G.D.D. & Wedell, N. 2008 Sex ratio distorter reduces sperm competitive ability in an insect. Evolution 62: 1644–1652. 18373627 10.1111/j.1558-5646.2008.00386.x

[jeb12792-bib-0039] Price, T.A.R. , Hurst, G.D.D. & Wedell, N. 2010 Polyandry prevents extinction. Curr. Biol. 20: 1–5. 20188561 10.1016/j.cub.2010.01.050

[jeb12792-bib-0040] Price, T.A.R. , Lewis, Z. , Smith, D.T. , Hurst, G.D.D. & Wedell, N. 2011 Remating in the laboratory reflects rates of polyandry in the wild. Anim. Behav. 82: 1381–1386.

[jeb12792-bib-0042] Price, T.A.R. , Lizé, A. , Marcello, M. & Bretman, A. 2012 Experience of mating rivals causes males to modulate sperm transfer in the fly *Drosophila pseudoobscura* . J. Ins. Physiol. 58: 1669–1675. 10.1016/j.jinsphys.2012.10.008 23085556

[jeb12792-bib-0043] Price, T.A.R. , Bretman, A. , Gradilla, A.C. , Reger, J. , Taylor, M.L. , Giraldo‐Perez, P. *et al* 2014 Does polyandry control population sex ratio via regulation of a selfish gene? Proc. R. Soc. Lond. B 281: 20133259. 10.1098/rspb.2013.3259 PMC3996604 24695427

[jeb12792-bib-0044] Rodríguez‐Muñoz, R. , Bretman, A. , Slate, J. , Walling, C. & Tregenza, T. 2010 Natural and sexual selection in a wild insect population. Science 328: 1269–1272. 20522773 10.1126/science.1188102

[jeb12792-bib-0045] Schnakenberg, S. , Matias, W. & Siegal, M. 2011 Sperm‐storage defects and live birth in *Drosophila* females lacking spermathecal secretory cells. PLoS Biol. 9: e1001192. 22087073 10.1371/journal.pbio.1001192 PMC3210755

[jeb12792-bib-0046] Schnakenberg, S. , Siegal, M. & Qazi, M. 2012 Oh, the places they'll go: Female sperm storage and sperm precedence in *Drosophila melanogaster* . Spermatogenesis 2: 224–235. 23087839 10.4161/spmg.21655 PMC3469444

[jeb12792-bib-0047] Shorrocks, B. 1972 Invertebrate Types: Drosophila. Ginn & Co, London.

[jeb12792-bib-0048] Simmons, L.W. 2001 Sperm Competition and Its Evolutionary Consequences in the Insects. Princeton University Press, Princeton.

[jeb12792-bib-0049] Simmons, L. & Fitzpatrick, J. 2012 Sperm wars and the evolution of male fertility. Reproduction 144: 519–534. 22984191 10.1530/REP-12-0285

[jeb12792-bib-0050] Snook, R.R. 2005 Sperm in competition: not playing by the numbers. Trends Ecol. Evol. 20: 46–53. 16701340 10.1016/j.tree.2004.10.011

[jeb12792-bib-0051] Snook, R.R. , Markow, T. & Karr, T. 1994 Functional nonequivalence of sperm in *Drosophila pseudoobscura* . Proc. Natl. Acad. Sci. USA 91: 11222–11226. 7972038 10.1073/pnas.91.23.11222 PMC45199

[jeb12792-bib-0052] Stern, C. & Hadorn, E. 1938 The determination of sterility in *Drosophila* males without a complete Y‐chromosome. Am. Naturalist 72: 42–45.

[jeb12792-bib-0053] Taylor, M. , Price, T. & Wedell, N. 2014 Polyandry in nature: a global analysis. Trends Ecol. Evol. 29: 376–383. 24831458 10.1016/j.tree.2014.04.005

[jeb12792-bib-0054] Uller, T. & Olsson, M. 2008 Multiple paternity in reptiles: patterns and processes. Mol. Ecol. 17: 2566–2580. 18452517 10.1111/j.1365-294X.2008.03772.x

[jeb12792-bib-0055] Voelker, R.A. & Kojima, K. 1972 Fertility and fitness of XO males in *Drosophila*. II. Quantiative analysis. Evolution 26: 560–573. 10.1111/j.1558-5646.1972.tb01964.x 28563346

[jeb12792-bib-0056] Wigby, S. , Sirot, L. , Linklater, J. , Buehner, N. , Calboli, F. , Bretman, A. *et al* 2009 Seminal fluid protein allocation and male reproductive success. Curr. Biol. 19: 751–757. 19361995 10.1016/j.cub.2009.03.036 PMC2737339

[jeb12792-bib-0057] Wolfner, M.F. 1997 Tokens of love: functions and regulation of *Drosophila* male accessory gland products. Ins. Biochem. Mol. Biol. 27: 179–192. 10.1016/s0965-1748(96)00084-7 9090115

[jeb12792-bib-0058] Wu, C.‐I. 1983 Virility deficiency and the *sex‐ratio* trait in *Drosophila pseudoobscura*. I. Sperm displacement and sexual selection. Genetics 105: 651–662. 17246170 10.1093/genetics/105.3.651 PMC1202179

