# Supplementary material for: Winter is coming: hibernation reverses the outcome of sperm competition in a fly
Source: J Evol Biol. 2015 Dec 28;29(2):371–9. doi: 10.1111/jeb.12792 (PMC4784169; doi:10.1111/jeb.12792)
Supplement: Supplementary file 1 — Table S1 Number of females surviving each temperature treatment, and the number that successfully produced offspring. [file JEB-29-371-s001.docx]

Supplementary Table S1

Number of females surviving each temperature treatment, and the number that successfully produced offspring.

Supplementary Table S1

| Days in cold | Females that survived cold treatment | Females that produced offspring | Proportion of females that produced offspring |
| --- | --- | --- | --- |
| 0 | 123 | 116 | 0.94 |
| 1 | 117 | 109 | 0.93 |
| 30 | 63 | 48 | 0.76 |
| 120 | 61 | 29 | 0.48 |
